# 

**Published:** 1871-06

**Authors:** 


### OUR EXCHANGES.

The following Journals have been received and entered upon our exchange list. These, with those previously noticed, embrace all the leading Journals published in the United States. We return our sincere thanks to the Proprietors and Editors of all, for the great kindness they have shown our Journal in so uniformly and generously placing it on their exchange lists.

The American Journal of Obstetrics, and Diseases of Women and Children: Edited by B. F. Dawson, M. D., Physician for Children at the New York Dispensation, etc., etc. Associate Editors: E. Noeggrath, M. D., late Professor of Obstetrics, and Diseases of Women and Children in the New York Medical College. A. Jacobi, M. D., Clinical Professor of Diseases of Children in the College of Physicians and Surgeons, New York. Published by Wm. Baldwin & Co., 21 Park Row, New York, at $5,00 per annum in advance. Specimen numbers, 75 cts. This valuable quarterly enters upon its fourth year. It is one of the very best Journals published in the country, devoted especially to Obstetrics and the Diseases of Women and Children. The May No. before us is worth, alone, the price of subscription, containing many original articles of great value to the practitioner.

The London Lancet: A Journal of British and Foreign Medicine, Physiology, Surgery, Chemistry, Criticism, Literature and News: Edited by James C. Wakley, M. D. M. R. C. S. Published Monthly. All subscriptions in advance, and must be ad: dressed to the agent, Wm. C. Herald, No. 52 John St., N. York. $5.00 per annum. It is only necessary to name the London Lancet to commend it. It should be in the hands of every physician in the country.

Oregon Medical and Surgical Reporter: Edited by E. R. Fiske, A. M. M. D., H. Carpenter, M. D., E. Y. Chase, M. D. Published quarterly, at Salem, Oregon, by Frank A. Cook, at $3.00 a year. This is a vigorous medical journal, and, if a true representative of the profession of the Pacific slope, speaks highly of their energy, learning and progress.

New York Medical Journal: Edited by Edward S. Dunster, M. D. Published by D. Appleton & Co., Nos. 549 and 551, Broadway, N. Y. $4.00 a year. This is a splendid monthly, both as to contents and typographical execution. The number before us contains several valuable original articles, the first in order being a highly interesting one on "Anæsthetics," from the pen of Ed. R. Squibb, M D., of Brooklyn, New York.

National Medical Journal, edited by S. C. Busey, M. D., and Wm. Lee, M. D. Published monthly by Judd & Detweiler, Washington, D. C. $3.00 a year. This Journal is ably conducted. Dr. Busey is a medical writer of singular force and ability.

The Northwestern Medical and Surgical Journal; Alexander M. Stone, M. D. Editor. Published monthly by the editor at St. Paul, Minn. $3.00 per annum. The number for May contains many original articles of value. It is one among the best of our Western exchanges.

The Medical Cosmos, a monthly Abstract of Medical Science and Art. Geo. J. Zeigler, M. D. Editor and Proprietor. Price $1.00 a year. The first number of the Cosmos was issued in May last. We wish it great success and prosperity.

Archives of Science and Transactions of the Orleans County Society of Natural Sciences. J. M. Currier, M. D. and G. A. Hinman, M. D. Editors. Published quarterly at Newport, Orleans county, Vt., at $2.50 a year. This Journal is scientific in its scope, and highly instructive.

The Druggists' Circular and Chemical Gazette: A Practical Journal of Chemistry, as applied to Pharmacy, Arts and Sciences, and General Business Organ for Druggists, Chemists and Apothecaries. L. V. Newton, M. D., Editor and Proprietor, 36 Beekman St., New York. $1.50 a year in advance. This Journal is a *sine qua non* to all druggists, and physicians will find it one of the best of advisers on many points in Pharmacy and Chemistry.

American Journal of Pharmacy, published by authority of the Philadelphia College of Pharmacy. Edited by John M. Maisch. $3.00 per annum in advance. This Journal "embodies a large amount of information, extremely valuable to Apothecaries, Druggists and Physicians," and should be in the hands of all these, being one of the best representatives of Pharmacy in this country.

The Eclectic Medical Journal of Pennsylvania, edited by John Buchanan, M. D,, $1.00 per annum. This Journal, as its name implies, is devoted to Eclecticism.

American Observer: a monthly journal devoted to the dissemination of Homœpathy: Ed. A. Lodge, M. D. General Editor; $2.00 per annum. Its title fully sets forth its objects. Published at Detroit, Mich.

---

### THE AMERICAN MEDICAL ASSOCIATION.

This body met in the city of San Francisco, Cal., according to appointment. Want of space will allow us simply to state that Dr. D. W. Yandell, of Louisville, Ky., was elected President. In the election of Dr. Yandell, we see the highest evidence of the kindly feelings of our Northern brethren towards their Southern brothers. This fact we are proud to chronicle. The next meeting of the Association will be held in Philadelphia, Pa., May 1872.

We hope to notice, at greater length the proceedings of the Association in our future numbers.

